# S-diclofenac Protects against Doxorubicin-Induced Cardiomyopathy in Mice via Ameliorating Cardiac Gap Junction Remodeling

**DOI:** 10.1371/journal.pone.0026441

**Published:** 2011-10-24

**Authors:** Huili Zhang, Alian Zhang, Changfa Guo, Chunzhi Shi, Yang Zhang, Qing Liu, Anna Sparatore, Changqian Wang

**Affiliations:** 1 Department of Cardiology, Shanghai Ninth People's Hospital, Shanghai JiaoTong University School of Medicine, Shanghai, China; 2 Department of Cardiac Surgery, Zhongshan Hospital, Fudan University, Shanghai, China; 3 Dipartimento di Scienze Farmaceutiche “Pietro Pratesi,” Università degli Studi di Milano, Milano, Italy; Brigham & Women's Hospital - Harvard Medical School, United States of America

## Abstract

Hydrogen sulfide (H_2_S), as a novel gaseous mediator, plays important roles in mammalian cardiovascular tissues. In the present study, we investigated the cardioprotective effect of S-diclofenac (2-[(2,6-dichlorophenyl)amino] benzeneacetic acid 4-(3H-1,2,dithiol-3-thione-5-yl)phenyl ester), a novel H_2_S-releasing derivative of diclofenac, in a murine model of doxorubicin-induced cardiomyopathy. After a single dose injection of doxorubicin (15 mg/kg, i.p.), male C57BL/6J mice were given daily treatment of S-diclofenac (25 and 50 µmol/kg, i.p.), diclofenac (25 and 50 µmol/kg, i.p.), NaHS (50 µmol/kg, i.p.), or same volume of vehicle. The cardioprotective effect of S-diclofenac was observed after 14 days. It showed that S-diclofenac, but not diclofenac, dose-dependently inhibited the doxorubicin-induced downregulation of cardiac gap junction proteins (connexin 43 and connexin 45) and thus reversed the remodeling of gap junctions in hearts. It also dose-dependently suppressed doxorubicin-induced activation of JNK in hearts. Furthermore, S-diclofenac produced a dose-dependent anti-inflammatory and anti-oxidative effect in this model. As a result, S-diclofenac significantly attenuated doxorubicin-related cardiac injury and cardiac dysfunction, and improved the survival rate of mice with doxorubicin-induced cardiomyopathy. These effects of S-diclofenac were mimicked in large part by NaHS. Therefore, we propose that H_2_S released from S-diclofenac *in vivo* contributes to the protective effect in doxorubicin-induced cardiomyopathy. These data also provide evidence for a critical role of H_2_S in the pathogenesis of doxorubicin-induced cardiomyopathy.

## Introduction

Doxorubicin is an anthracycline antibiotic widely used in the treatment of a variety of human neoplastic diseases and solid cancers, but its clinical application is hampered by progressive and does-related cardiotoxicity including transient electrocardiographic abnormalities, cardiomyopathy and heart failure. The efficacy of doxorubicin as a cytotoxic agent for the treatment of human tumors has prompted a search to develop new agents to reduce its cardiotoxicity [1]–[5].

Hydrogen sulfide, as a novel gaseous mediator, is generated endogenously during cysteine metabolism in many types of mammalian cells in the reaction catalysed by cystathionine β-synthase (CBS) and cystathionine γ-lyase (CSE) [6]. Although CBS and CSE are widely distributed in tissues, CBS is the main H_2_S-forming enzyme in central nervous system while CSE is the major H_2_S-producing enzyme in cardiovascular system. It has become clear that H_2_S fulfills a wide range of physiological and pathological functions in cardiovascular system [6]. Recent study has shown that deficiencies of H_2_S synthesis may contribute to the pathogenesis of doxorubicin-induced cardiomypathy and that administration of NaHS, an H_2_S donor ameliorated doxorubicin-related cardiac dysfunction via inhibiting oxidative stress injury [7]. Therefore, application of H_2_S donors may be a promising therapeutic strategy to prevent doxorubicin cardiotoxicity. However, NaHS is a short-lasting H_2_S donor, and it releases H_2_S quickly, which may cause an acute alteration in hemodynamics. As a result, further studies are needed to develop novel and potent compounds that are capable of producing H_2_S slowly and stably.

S-diclofenac (2-[(2,6-dichlorophenyl) amino] benzene acetic acid 4-(3H-1,2,dithiol-3-thione-5-yl) phenyl ester) is a novel molecule comprising a H_2_S-releasing dithiol-thione moiety attached by an ester linkage to diclofenac. Unlike other H_2_S donors, S-diclofenac releases H_2_S slowly both in vitro and in vivo over a period of several hours [8], [9]. Furthermore, S-diclofenac has comparable or even improved anti-inflammatory property while its gastrointestinal-damaging effect is considerably reduced as compared with the parent drug [8], [9]. These findings suggest that S-diclofenac may be an attractive alternative to the available H_2_S donors. On the other hand, one recent study has shown that S-diclofenac could protect against ischemia–reperfusion injury in the isolated rabbit heart [10]. The experiment, which indicates the beneficial effect of S-diclofenac in ischemic heart diseases, prompted us to investigate the possible protective effect of S-diclofenac against non-ischemic origin. We hypothesized that S-diclofenac might be able to block the progression of cardiomyopathy induced by doxorubicin, known for its late-onset acute and chronic cardiotoxicity. To test that idea, in the present study, we examined the cardioprotective effect of S-diclofenac in a murine model of doxorubicin-induced cardiomyopathy and investigated the specific mechanisms of its effect. Our data suggests that S-diclofenac may attenuate the cardiotoxicity of doxorubicin by reversing cardiac gap junction remodeling along with inhibiting oxidative injury and inflammation in hearts.

## Results

### Deficiencies of H_2_S formation in doxorubicin-induced cardiomyopathy

The model of dilated cardiomyopathy was induced in male 10-week-old C57BL/6J mice by a single intraperitoneal injection of doxorubicin at a dose of 15 mg/kg. The success of the model was confirmed by decreased LV function and enlarged left ventricle of the mice (by echocardiography). At necropsy, those mice exhibited ascites and lobulated enlarged livers, indicative of liver congestion, or foamy lungs indicative of lung edema, suggesting that they undergo congestive heart failure.

**Figure 1:**
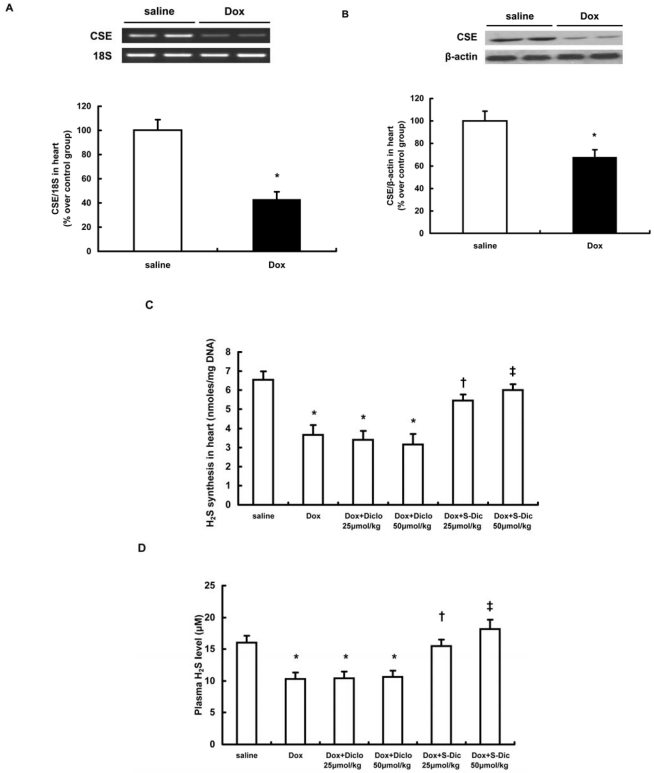
Alterations in endogenous H_2_S synthesis 14 days after doxorubicin injection (A–D) and effect of S-diclofenac on the synthesis of H_2_S in doxorubicin-induced cardiomyopathy (C, D). Male 10-week-old C57BL/6J mice were randomly given doxorubicin (15 mg/kg, i.p.) or same volume of saline. Treatment with S-diclofenac (25 and 50 µmol/kg, i.p.) or diclofenac (25 and 50 µmol/kg, i.p.) started 2 hours after doxorubicin injection and continued every 24 hours. Fourteen days after doxorubicin injection, the expression level of CSE mRNA (A) and protein in hearts (B), H_2_S synthesizing activity in hearts (C) and plasma H_2_S concentration (D) were measured as described in Materials and Methods. Results shown are the mean ± SEM (n = 6–8 animals in each group). * P<0.01 vs saline group; † P<0.05 vs Dox+Diclo 25 µmol/kg group (animals treated with doxorubicin+diclofenac 25 µmol/kg); ‡ P<0.01 vs Dox+Diclo 50 µmol/kg group (animals treated with doxorubicin+diclofenac 50 µmol/kg).

Administration of doxorubicin resulted in a significant down-regulation of CSE expression in heart. The mRNA and protein levels of CSE were significantly decreased in hearts 14 days after doxorubicin injection (n = 6–8 in each group, P<0.01, Fig. 1A and 1B). As a result, a remarkable reduction in cardiac H_2_S synthesizing activity and plasma level of H_2_S was observed in doxorubicin injection group (n = 6–8 in each group, P<0.01, Figure 1C and 1D). Not surprisingly, consecutive administration with S-diclofenac for 14 days dose-dependently diminished the deficiencies of H_2_S formation induced by doxorubicin. As shown in Figure 1C and 1D, S-diclofenac, but not diclofenac dose-dependently abolished doxorubicin-induced reduction in cardiac H_2_S synthesizing activity and plasma H_2_S concentration.

### Alterations of gap junction proteins in doxorubicin-induced cardiomyopathy

**Figure 2:**
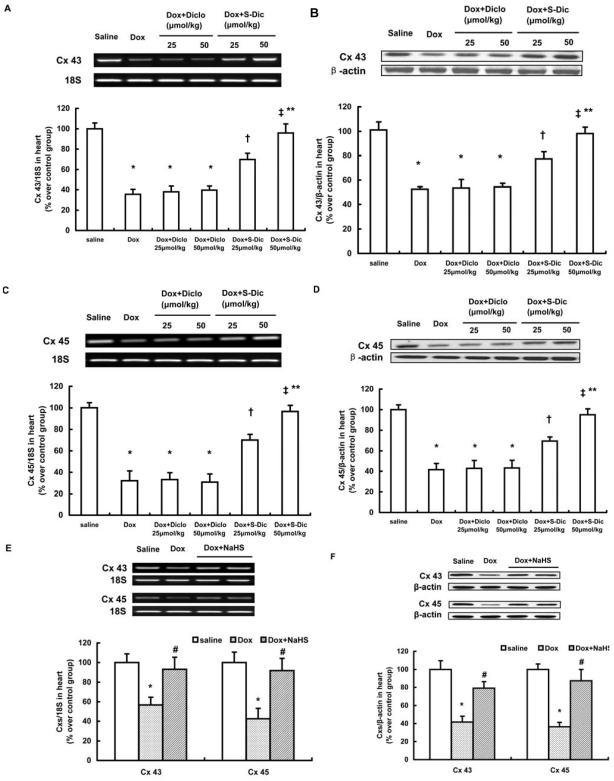
Effect of treatment with S-diclofenac (A–D) or NaHS (E, F) on Cx43 and Cx45 expression in hearts 14 days after doxorubicin injection. Male 10-week-old C57BL/6J mice were randomly given doxorubicin (15 mg/kg, i.p.) or same volume of saline. Treatment with S-diclofenac (25 and 50 µmol/kg, i.p.), diclofenac (25 and 50 µmol/kg, i.p.), NaHS (50 µmol/kg, i.p.) or vehicle started 2 hours after doxorubicin injection and continued every 24 hours. Fourteen days after doxorubicin injection, hearts were harvested and examined by RT-PCR and Western blot for the expression levels of Cx43 and Cx45. After analysis by densitometry, the data were expressed as ratios of Cxs to 18S or Cxs to β-actin (plotted as percentage over saline group). Results shown are the mean ± SEM (n = 6–8 animals in each group). * P<0.05 vs saline group; † P<0.05 vs Dox+Diclo 25 µmol/kg group; ‡ P<0.05 vs Dox+Diclo 50 µmol/kg group; ** P<0.05 vs. Dox+S-Dic 25 µmol/kg group (animals treated with doxorubicin and S-diclofenac 25 µmol/kg); # P<0.05 vs. Dox group.

Gap junction channels are formed by the association of two connexons composed of six protein subunits termed connexins (Cxs) [11], [12]. In mammalian hearts, gap junctions are principally composed of three major connexin isoforms, Cx43, Cx40 and Cx45. Therefore, we examined the cardiac expression levels of Cx43, Cx45 and Cx40 in doxorubicin-induced cardiomyopathy. As shown in Figure 2, the mRNA and proteins levels of Cx43 and Cx45 in animal hearts were drastically downregulated 14 days after doxorubicin injection (n = 6–8 in each group, P<0.01). However, no significant change in Cx40 mRNA and protein expression was observed in animals injected with doxorubicin (data not shown). It may be due to the fact that of most mammalian species, Cx40 is abundant in gap junctions of atrial myocytes but not of working ventricular myocytes [11], [12].

### Effect of S-diclofenac on doxorubicin-induced downregulation of gap junction proteins

S-diclofenac at doses of 25 or 50 µmol/kg significantly alleviated doxorubicin-induced downregulation of Cx43 and Cx 45, as compared to a mole for mole equivalent dose of diclofenac (n = 6–8 in each group, P<0.05, Figure 2A–D). S-diclofenac at a higher dose was more potent than that at a lower dose to reverse the suppression of Cxs expression in doxorubicin-induced cardiomypathy. Furthermore, the effect of S-diclofenac on gap junction proteins was in large part resembled by treatment with NaHS, a conventional H_2_S donor. As shown in Figure 2E and 2F, NaHS at a dose of 50 µmol/kg prominently provoked the mRNA and protein expressions of Cx43 and Cx45 in doxorubicin-injected mice (n = 6–8 in each group, P<0.05).

### Effect of S-diclofenac on doxorubicin-induced gap junction remodeling

**Figure 3:**
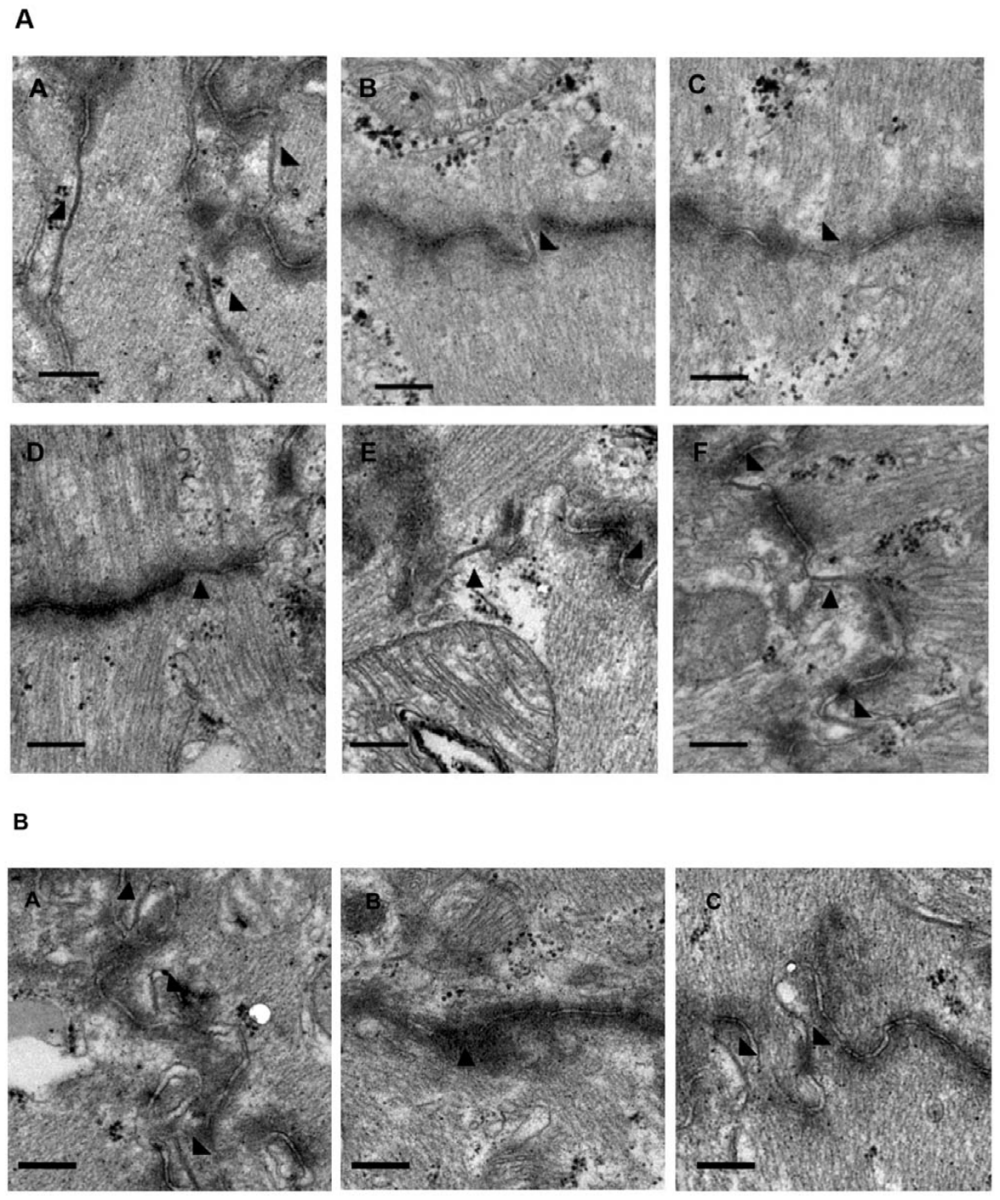
Effect of treatment with S-diclofenac (A) or NaHS (B) on gap junction remodeling in hearts 14 days after doxorubicin injection. (A) Representative transmission electron micrographs of gap junctions in hearts from saline group (A), Dox group (B), Dox+Diclo 25 µmol/kg group (C), Dox+Diclo 50 µmol/kg group (D), Dox+S-Dic 25 µmol/kg group (E) and Dox+S-Dic 50 µmol/kg group (F) (n = 5 animals in each group). (B) Representative transmission electron micrographs of gap junctions in hearts from saline group (A), Dox group (B), Dox+NaHS 50 µmol/kg group (C). Gap junctions are identified by **arrows**. Bars represent 0.2 µm (original magnification: ×30000).

Doxorubicin-associated gap junction remodeling in mouse heart was examined by transmission electron microscopy. Ultrastructural morphometry showed a significant reduction in gap junction density (number of gap junctions per unit intercalated disk length) 14 days after doxorubicin injection (Figure 3A and Table 1). Aggregate gap junction length per unit intercalated disk length was also declined in doxorubicin group. Treatment with S-diclofenac but not diclofenac led to a remarkable increase in both the number and length of cardiomyocyte gap junctions (Figure 3A and Table 1). A higher dose of S-diclofenac resulted in a more impressive rise in gap junction number and length as compared to a lower dose (Figure 3A and Table 1). Similarly, NaHS significantly reversed the doxorubicin-induced gap junction remodeling in heart (Figure 3B and Table 2).

**Table 1:** Effect of S-diclofenac on gap junction remodeling induced by doxorubicin.

	Number of gap junctions	Gap junction length
	(per 100 µm ID length)	(µm per 100 µm ID length)
Saline	19.33±4.63	17.67±4.40
Dox	4.57±2.69*	5.50±2.35*
Dox+Diclo 25 µmol/kg	4.85±2.79*	5.96±2.16*
Dox+Diclo 50 µmol/kg	5.14±1.57*	6.23±1.96*
Dox+S-Dic 25 µmol/kg	9.85±2.41†	10.81±2.04†
Dox+S-Dic 50 µmol/kg	14.57±2.63‡ **	15.35±2.83‡ **

*P<0.01 vs saline group;

† P<0.05 vs mice treated with doxorubicin+diclofenac 25 µmol/kg;

‡ P<0.05 vs mice treated with doxorubicin+diclofenac 50 µmol/kg;

**P<0.05 vs mice treated with doxorubicin+S-diclofenac 25 µmol/kg; ID: intercalated disc.

**Table 2:** Effect of NaHS on gap junction remodeling induced by doxorubicin.

	Number of gap junctions	Gap junction length
	(per 100 µm ID length)	(µm per 100 µm ID length)
saline	18.17±7.41	15.57±3.65
Dox	5.29±2.87*	6.71±1.53*
Dox+NaHS	12.14±2.97**	10.26±2.06**

*P<0.05 vs saline group;

**P<0.05 vs mice treated doxorubicin.

### Effect of S-diclofenac on doxorubicin-induced JNK activation in hearts

**Figure 4:**
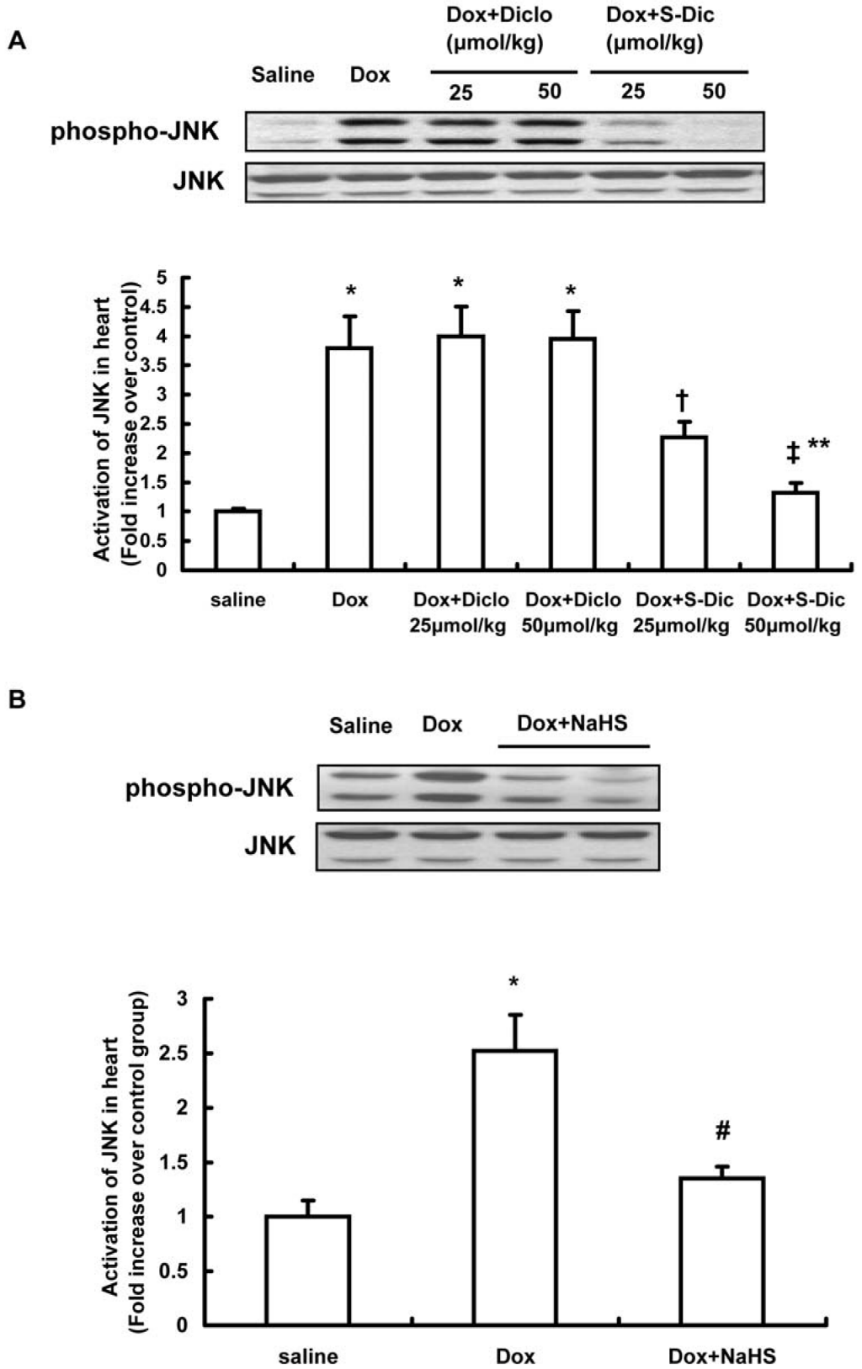
Effect of treatment with S-diclofenac (A) or NaHS (B) on JNK activation in hearts 14 days after doxorubicin injection. Male 10-week-old C57BL/6J mice were randomly given doxorubicin (15 mg/kg, i.p.) or same volume of saline. Treatment with S-diclofenac (25 and 50 µmol/kg, i.p.), diclofenac (25 and 50 µmol/kg, i.p.) or NaHS (50 µmol/kg, i.p.) started 2 hours after doxorubicin injection and continued every 24 hours. Fourteen days after doxorubicin injection, hearts were harvested and examined by Western blot for total and phospho-JNK. After analysis by densitometry, the data were expressed as ratios of phosphorylated protein to total protein (plotted as fold increase over saline group, n = 6 animals in each group). * P<0.05 vs saline group; † P<0.05 vs Dox+Diclo 25 µmol/kg group; ‡ P<0.05 vs Dox+Diclo 50 µmol/kg group; ** P<0.05 vs. Dox+S-Dic 25 µmol/kg group; # P<0.05 vs. Dox group.

To get more insights into the intracellular mechanism by which S-diclofenac upregulated the cardiac expression of connexins in doxorubicin-induced cardiomyopathy, further experiments were performed to investigate the effect of S-diclofenac on JNK activation, known to play a part in modulating the connexin expression [13]–[16]. It was found that administration of doxorubicin significantly activated the phosphorylation of JNK in hearts (Figure 4, n = 6 in each group). Treatment with S-diclofenac dose-dependently inhibited doxorubicin-induced activation of JNK (n = 6 in each group, Figure 4A). NaHS at a dose of 50 µmol/kg also markedly blocked the phosphorylation of JNK in doxorubicin-injected mice (n = 6 in each group, P<0.05, Figure 4B). In contrast, diclofenac had no effect on JNK activation (Figure 4A). In addition, we also examined the effect of H_2_S on ERK1/2 and p38 MAPK pathways in this animal model. Both S-diclofenac and NaHS failed to affect doxorubicin-evoked activation of ERK1/2 and p38 in hearts (data not shown).

### Effect of S-diclofenac on doxorubicin-induced inflammation and oxidative stress

Overproduction and/or ineffective scavenging of reactive oxygen species (ROS) as well as inflammation play a crucial role in the pathogenesis of doxorubicin-induced cardiomyopathy [2], [3]. Therefore, we examined the effect of S-diclofenac on lipid antioxidative mechanism (SOD and GSH activity), lipid peroxidation activity (MDA content), and neutrophil infiltration (MPO activtity) in hearts. As compared to a mole for mole equivalent dose of diclofenac, S-diclofenac more potently alleviated doxorubicin-induced inflammation in hearts as evidenced by a distinct reduction in cardiac MPO activity (Table 3). Consistently, the severity of oxidative stress caused by doxorubicin was also significantly reduced by treatment with S-diclofenac. As shown in Table 3, S-diclofenac, but not its parent drug markedly elevated the cardiac activity of SOD and GSH-px and decreased the level of MDA in hearts. The anti-inflammatory and anti-oxidative effect of S-diclofenac was more potent at a higher dose. In addition, treatment with NaHS also significantly inhibited doxorubicin-associated inflammation and oxidative damage (Table 4).

**Table 3:** Effect of S-diclofenac on inflammatory response and oxidative stress in heart.

	MPO activity	MDA	SOD	GSH-Px
	(Fold increase over control)	(nmol/g protein)	(U/mg protein)	(U/mg protein)
Saline	1±0.20	73.99±3.64	137.46±10.38	34.09±2.68
Dox	2.89±0.69*	161.05±5.32*	70.51±6.94*	16.41±1.18*
Dox+Diclo 25 µmol/kg	2.31±0.35* #	158.23±7.25*	68.46±5.97*	15.49±1.24*
Dox+Diclo 50 µmol/kg	2.08±0.20* #	163.77±9.83*	65.94±4.38*	14.71±1.54*
Dox+S-Dic 25 µmol/kg	1.70±0.33†	127.43±6.77†	97.85±3.41†	22.98±1.23†
Dox+S-Dic 50 µmol/kg	1.15±0.24‡ **	90.90±10.28‡ **	124.63±7.09‡ **	30.29±1.98‡ **

*P<0.01 vs saline group;

# P<0.05 vs mice treated with doxorubicin;

† P<0.05 vs mice treated with doxorubicin+diclofenac 25 µmol/kg;

‡ P<0.05 vs mice treated with doxorubicin+diclofenac 50 µmol/kg;

**P<0.05 vs mice treated with doxorubicin+S-diclofenac 25 µmol/kg.

**Table 4:** Effect of NaHS on inflammatory response and oxidative stress in heart.

	MPO activity	MDA	SOD	GSH-Px
	(Fold increase over control)	(nmol/g protein)	(U/mg protein)	(U/mg protein)
saline	1±0.16	82.26±4.36	147.45±11.16	36.11±1.89
Dox	2.46±0.46*	134.01±6.23*	78.26±4.89*	13.08±1.04*
Dox+NaHS	1.46±0.27**	102.36±9.55**	109.83±4.65**	19.08±1.81**

* P<0.05 vs saline group;

** P<0.05 vs mice treated doxorubicin.

### Effect of S-diclofenac on doxorubicin-induced cardiac injury and cardiac dysfunction

**Figure 5:**
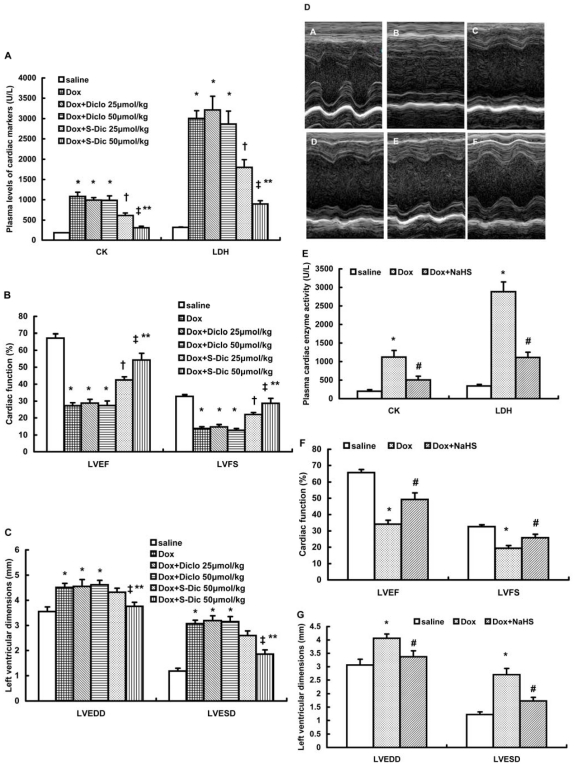
Effect of treatment with S-diclofenac or NaHS on plasma levels of cardiac marker enzymes and cardiac function 14 days after doxorubicin injection. Male 10-week-old C57BL/6J mice were randomly given doxorubicin (15 mg/kg, i.p.) or same volume of saline. Treatment with S-diclofenac (25 and 50 µmol/kg, i.p.). diclofenac (25 and 50 µmol/kg, i.p.) or NaHS (50 µmol/kg, i.p.) started 2 hours after doxorubicin injection and continued every 24 hours. Fourteen days after doxorubicin injection, plasma activities of CK and LDH (A, E), left ventricular systolic function (B, F) as well as left ventricular dimensions (C, G) were measured as described in Materials and Methods (n = 6–8 animals in each group). (D) Representative M-mode echocardiographs from saline group (A), Dox group (B), Dox+Diclo 25 µmol/kg group (C), Dox+Diclo 50 µmol/kg group (D), Dox+S-Dic 25 µmol/kg group (E) and Dox+S-Dic 50 µmol/kg group (F). * P<0.05 vs saline group; † P<0.05 vs Dox+Diclo 25 µmol/kg group; ‡ P<0.05 vs Dox+Diclo 50 µmol/kg group; ** P<0.05 vs. Dox+S-Dic 25 µmol/kg group; # P<0.05 vs. Dox group.

Evidence of cardiac injury induced by intraperitoneal administration of doxorubicin was indicated by an obvious increase in plasma levels of cardiac markers (LDH and CK) (Figure 5A), an evident depression in left ventricular systolic function (LVEF and LVFS) (Figure 5B) and an apparent dilation of left ventricles (Figure 5C). Treatment with S-diclofenac but not diclofenac protected mice against doxorubicin-induced cardiac injury. As shown in figure 5A, plasma activities of LDH and CK were significantly declined in mice treated with doxorubicin and S-diclofenac as compared to those treated with doxorubicin and diclofenac. The impairment of cardiac function caused by doxorubicin was also attenuated by treatment with S-diclofenac, as characterized by a significant elevation in LVEF and LVFS (n = 6–8 in each group, P<0.05, Figure 5B, 5D). S-diclofenac at a dose of 50 µmol/kg, but not at a lower dose notably repressed the enlargement of left ventricle induced by doxorubicin (n = 6–8 in each group, P<0.05, Figure 5C, 5D). Similarly, NaHS significantly decreased the plasma levels of LDH and CK, improved the left ventricular dysfunction and attenuated left ventricle dilation in doxorubicin-injected mice (n = 6–8 in each group, Figure 5E, 5F, 5G).

**Figure 6:**
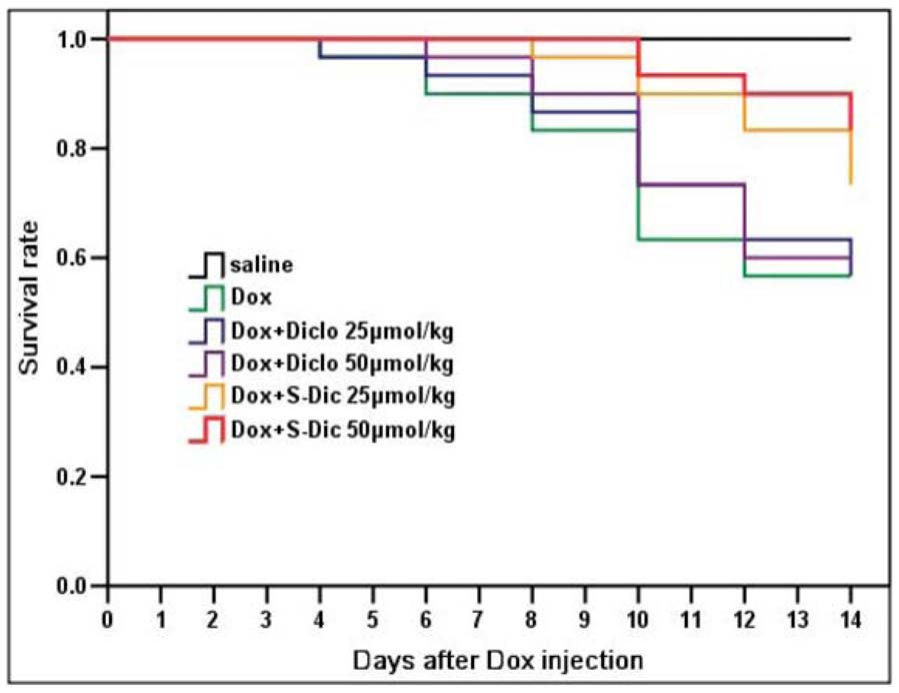
Effect of treatment with S-diclofenac on survival rate of mice subjected to doxorubicin injection. Male 10-week-old C57BL/6J mice were randomly given doxorubicin (15 mg/kg, i.p.) or same volume of saline. Treatment with S-diclofenac (25 and 50 µmol/kg, i.p.) or diclofenac (25 and 50 µmol/kg, i.p.) started 2 hours after doxorubicin injection and continued every 24 hours. Their survival rate was monitored for 14 days (n = 20 animals in each group). The 14-day survival rate in Dox+S-Dic 50 µmol/kg group were significantly higher than that in Dox+Diclo 50 µmol/kg group (P<0.05).

In addition, administration with doxorubicin in mice resulted in a high 14-day mortality rate. Treatment with S-diclofenac but not diclofenac significantly improved the survival rate of the mice with doxorubicin-induced cardiomyopathy. The 14-day survival rate of the mice with doxorubicin-induced cardiomyopathy was significantly elevated when treated with S-diclofenac at a dose of 50 µmol/kg (n = 20 in each group, P<0.05, Figure 6).

## Discussion

In the present study, we found that doxorubicin injection downregulated the cardiac expression of CSE along with reduced plasma H_2_S level and cardiac H_2_S synthesizing activity. Our findings are in accord with earlier observations and reinforce the significance of H_2_S insufficiency in the development of doxorubicin-induced cardiotoxicity [7]. These results provide a solid basis for the use of H_2_S donor drugs in the treatment of doxorubicin-induced cardiotoxicity. We showed here that S-diclofenac, an H_2_S-releasing diclofenac derivative, provided marked cardioprotection in myocardial injury and cardiac dysfunction resulting from doxorubicin injection. The cardioprotective effect of S-diclofenac could be mimicked in large part by a mole for mole equivalent dose of NaHS, a conventional H_2_S donor. Our findings suggest that H_2_S release (from S-diclofenac) protects animals against doxorubicin-associated cardiotoxicity and improves the outcome of animals with doxorubicin-induced cardiomyopathy.

Although the pathogenesis of anthracycline cardiotoxicity has been under intensive investigation for many years, the underlying mechanisms are still not fully understood. Multiple explanations have been proposed for the doxorubicin cardiotoxicity, e.g. oxidative stress, inflammation, DNA damage, attenuation in protein synthesis, alterations in cellular energetics and cardiomyocyte apoptosis [1]–[5]. However, the present study is focused on three aspects of the action of doxorubicin with respect to gap junction remodeling, oxidative injury and inflammation.

Gap junction channels are responsible for direct cell-to-cell communication and remodeling of gap junction organization is a conspicuous feature of heart diseases. In the present study, we have demonstrated for the first time that doxorubicin suppressed cardiac expression of Cx43 and Cx45, resulting in loss of Cx43/Cx45 junction channels and consequent spatial remodeling of gap junctions, and thereby leading to progressive cardiac dysfunction and left ventricular remodeling. Treatment with S-diclofenac, but not diclofenac, not only remarkably upregulated the cardiac expression of Cxs (Cx43 and Cx45) mRNA and proteins, but also raised the density and length of gap junctions in doxorubicin-induced cardiomyopathy. As a result, it considerably attenuated heart injury and left ventricular enlargement, and improved left ventricular ejection function and the survival rate of animals with doxorubicin-induced cardiomyopathy. Moreover, the protective effect of S-diclofenac could be mimicked by NaHS. Our data straightforwardly demonstrate that H_2_S may exert its cardioprotective effect in doxorubicin-induced cardiomyopathy via regulating connexin expression.

JNK is a crucial regulator of AP-1 activation which has been shown to be necessary for activating Cx43 and Cx45 proximal promoter [13], [14]. Activation of JNK induced significant loss of Cx43 expression and decreased the numbers of gap junctions in cultured cardiomyocytes and transgenic mouse ventricular myocardium [15], [16]. Therefore, we further examined the relevance of JNK pathway to the protective effect of S-diclofenac. We found that doxorubicin induced an obvious activation of JNK in hearts, suggesting that doxorubicin-induced downregulation of Cx43 and Cx45 is possibly mediated by JNK pathway. Treatment with S-diclofenac or NaHS significantly inhibited the phosphorylation of JNK in hearts from doxorubicin-injected mice. Hence, it seems reasonable to assume that S-diclofenac derived H_2_S may abolish doxorubicin-associated downregulation of Cxs by inhibiting the cardiac activity of JNK. However, the precise points of interaction between S-diclofenac and JNK pathway can not be determined in the present in vivo experiments and further work is needed most likely in an isolated cardiomyocyte system to probe the exact mechanisms involved. In addition to transcription, post-translational modifications such as phosphorylation are also important for connexin trafficking and assembly at gap junctions [17], [18]. It has been shown that phosphorylation of connexins regulates many aspects of the gap junction life cycle including assembly, gating, internalization, and degradation [17]. Whether H_2_S could alter the biological functions of gap junctions via modulating the phosphorylation of Cxs in doxorubicin-induced cardiotoxicity will be the subject of our future study.

Moreover, latest studies have proposed that doxorubicin-induced cardiomyopathy is primarily a stem cell disease [19], [20]. Cardiotoxicity of the anthracycline is not restricted to cardiomyocytes but affects resident cardiac progenitor cells (CPCs) even more dramatically. Anthracycline not only inhibited the growth and survival of CPCs but also impaired global gene expression of CPCs. Reduction in the number of CPCs and defects in progenitor cell function finally lead to the development of cardiac myopathy or result in an adult heart that is more susceptible to stress. These findings offer an original possibility that H_2_S or H_2_S donor drugs may attenuate the loss of CPCs or the impairment of CPC capacity induced by doxorubicin, thus enhancing the myocardial regeneration and restoring the structural integrity of cardiomyopathic heart. Interestingly, in the present study, we did find that S-diclofenac or NaHS significantly elevated the expression levels of junctional proteins (Cx43 and Cx45) and partially reversed the remodeling of gap junctions in hearts. Therefore, it is meaningful to further investigate the type of cardiac cells affected by H_2_S and the underlying molecular mechanisms in the future study.

On the other hand, we found that doxorubicin suppressed the activities of antioxidant enzymes and caused overproduction and/or ineffective scavenging of ROS in hearts while S-diclofenac prevented these pathological processes. Similar antioxidative properties of H_2_S or H_2_S donor drugs were observed previously in neurons, vascular smooth muscle cells and neutrophils. H_2_S may suppress ROS generation and ameliorate oxidative insult by different mechanisms: 1) H_2_S may increase the activity of diverse antioxidant enzymes; 2) H_2_S may increase the levels of glutathione participating in regulating oxidative stress; 3) H_2_S, as a highly reactive molecule may easily react with ROS and/or reactive nitrogen species (NOS); 4) H_2_S may open ATP-dependent K^+^ and Cl^−^ channels [21]–[23]. Additionally, S-diclofenac has been shown to exhibit greater anti-inflammatory activity (compared to diclofenac) in the present study. A superior anti-inflammatory effect of S-diclofenac (compared to diclofenac) was also observed in animal models of hindpaw edema, acute panreatitis as well as endotoxemia [8], [24], [25]. The additional anti-inflammatory activity of S-diclofenac may be at least partially due to release of H_2_S. S-diclofenac derived H_2_S inhibited inflammatory response by downregulating the transcription of inflammation related genes via NF-κB pathway [8]. However, it is to be noted that H_2_S has been shown to play complex but poorly understood roles in inflammation with evidence for proinflammatory as well as anti-inflammatory properties. It was reported that H_2_S provoked the inflammatory response in sepsis, acute pancreatitis and endotoxemia [26], [27], [28]. In contrast, H_2_S inhibited leukocyte migration in animal models of paw edema and nonsteroidal anti-inflammatory drugs (NSAIDs)-induced gastric injury [29], [30]. Although the role of H_2_S in inflammation remains controversial, it raises the possibility that H_2_S may act as a double-edged sword in inflammatory conditions. It may act as an anti-inflammatory mediator to at physiological concentration, but may contribute to inflammation in an already inflamed area when overproduced.

In addition, S-diclofenac has distinct advantages over NaHS in the treatment of doxorubicin-induced cardiomyopathy, although both of them protected animals against doxorubicin-induced myocardial injury. Firstly, H_2_S release from S-diclofenac is a slow process probably mediated by plasma/tissue esterase, which split the ester linkage of the parent compound to form ADT-OH. This molecule thereafter releases H_2_S either by spontaneous chemical degradation or by an as yet unidentified metabolic process. Hence, S-diclofenac has been considered as a slow-releasing H_2_S donor. Administration of S-diclofenac had minimal effect on blood pressure [8]. In stark contrast, NaHS breaks down rapidly in aqueous solution to yield Na^+^ and HS^−^, the latter then reacting with H^+^ to form H_2_S. Administration of NaHS caused a pronounced but transient decrease in blood pressure [28], [31]. Secondly, recent studies have demonstrated that S-diclofenac may have a potential usefulness in cancer treatment [32]. S-diclofenac has been found to inhibit the activity and expression of carcinogen activating enzymes and may serve as effective chemoprevention agents by balance the equation of carcinogen activation and detoxification mechanisms [32]. Therefore, we propose that in comparison to NaHS, S-diclofenac may be a better option to prevent cardiac complications from anthracyclines in cancer patients undergoing chemotherapy with doxorubicin.

In summary, S-diclofenac attenuated doxorubicin-induced cardiac gap junction remodeling, oxidative stress and inflammatory response. The pharmacological profiles of S-diclofenanc, i.e. cardioprotective activity, suggest that this compound might constitute a potentially new therapeutic option to prevent doxorubicin-induced cardiotoxicity. However, further clinical studies will have to verify if this concept is valid in patients as well, and whether or not S-diclofenac treatment counteracts with the oncological effect of doxorubicin.

## Materials and Methods

### Chemicals

Doxorubicin was obtained from Wanle Pharmaceutical Industry (Shenzhen, China) as a 10 mg/bottle lyophilized powder. The malondialdehyde (MDA, a marker of lipid peroxidation and free radical activity), glutathione peroxidase (GSH-Px) and superoxide dismutase (SOD) assay kits were purchased from Nanjing Jiancheng Bio-engineering Institute (Nanjing, China). S-diclofenac was obtained from CTG Pharma, Milan, Italy.

### Animals and experimental protocols

The study was carried out in strict accordance with the recommendations in the Guide for the Care and Use of Laboratory Animals of the Shanghai JiaoTong University School of Medicine. The protocol was approved by the Committee on the Ethics of Animal Experiments of the Shanghai JiaoTong University School of Medicine (Permit Number: [2011]-22). Male 10-week-old C57BL/6J mice were randomly assigned to receive doxorubicin (15 mg/kg, i.p.) or same volume of saline (i.p.). Two hours after doxorubicin injection, diclofenac (25 and 50 µmol/kg, i.p.), S-diclofenac (25 and 50 µmol/kg, i.p.), NaHS (50 µmol/kg, i.p.) or same volume of vehicle (0.5% w/v carboxymethylcellulose or saline, i.p.) were administered daily for 14 consecutive days. The dosage of S-diclofenac was determined based on previous study [8]. Fourteen days after doxorubicin injection, animals were sacrificed by an i.p. injection of a lethal dose of pentobarbitone (90 mg/kg). Samples of hearts and plasma were harvested and stored at −80°C for subsequent measurement.

### Measurement of plasma H_2_S

Aliquots (120 µl) of plasma were mixed with distilled water (100 µl), trichloroacetic acid (10% w/v, 120 µl), zinc acetate (1% w/v, 60 µl), N, N-dimethyl-p-phenylenediamine sulfate (20 µM; 40 µl) in 7.2 M HCl and FeCl_3_ (30 µM; 40 µl) in 1.2 M HCl. The absorbance of the resulting solution (670 nm) was measured 10 min thereafter by spectrophotometry (Tecan Systems Inc.). H_2_S was calculated against a calibration curve of sodium hydrosulfide (NaHS; 3.125–100 µM). Results were showed plasma H_2_S concentration in µM.

### Assay of H_2_S synthesizing activity

H_2_S synthesizing activity in heart homogenates was measured essentially as described elsewhere [26]. Briefly, the assay mixture contained 100 mM potassium phosphate buffer (pH 7.4), L-cysteine (20 µl, 20 mM), pyridoxyal 5′-phosphate (20 µl, 2 mM), saline (30 µl) and 4.5% w/v tissue homogenate (430 µl). The reaction was performed in tightly sealed micro centrifuge tubes and initiated by transferring the tubes from ice to a water bath at 37°C. After incubation for 30 min, 1% w/v zinc acetate (250 µl) was added to trap evolved H_2_S followed by 10% v/v trichloroacetic acid (250 µl) to denature the protein and stop the reaction. Subsequently, N, N-dimethyl-p-phenylenediamine sulfate (20 µM; 133 µl) in 7.2 M HCl was added, immediately followed by FeCl3 (30 µM; 133 µl) in 1.2 M HCl. The absorbance of the resulting solution at 670 nm was measured by spectrophotometry (Tecan Systems Inc). The H_2_S concentration was calculated against a calibration curve of NaHS. Results were then corrected for the DNA content of the tissue sample [33] and were expressed as nmoles H_2_S formed/mg DNA.

### RT-PCR analysis

Total RNA from heart was extracted with Trizol ® reagent (Invitrogen, Carlsbad, CA, USA) according to the manufacturer's protocol. The concentration of isolated nuclei acids was determined spectrophotometrically by measuring the absorbance at 260 nm and the integrity was verified by ethidium bromide staining of 18S and 28S rRNA bands on a denaturing agarose gel. All samples were thereafter stored at −80°C until required. 1 µg RNA was reversely transcribed using iScript™ cDNA Synthesis Kit (Biorad, Hercules, CA, USA) at 25°C for 5 minutes, 42°C for 30 minutes, followed by 85°C for 5 minutes. The cDNA was used as a template for PCR amplification by iQ™ Supermix (Biorad, Hercules, CA, USA). The primer sequences for detection of CSE, Cx43, Cx45, Cx40 and 18S, optimal annealing temperature, optimal cycles and product sizes were as shown in table 5. PCR amplification was carried out in MyCycler™ (Biorad, Hercules, CA, USA). The reaction mixture was first subjected to 95°C for 3 minutes, followed by an optimal cycle of amplifications, consisting of 95°C for 50 s, optimal annealing temperature for 45 s and 72°C for 1 minute. PCR products were analyzed on 1.5% w/v agarose gels containing 0.5 µg/ml ethidium bromide.

**Table 5:** PCR primer sequences, optimal conditions and product sizes.

Gene	Primer sequence	Optimal conditions	Size
CSE	Forward: 5′-GACCTCAATAGTCGGCTTCGTTTC-3′	34 cycles	618 bp
	Reverse: 5′-CAGTTCTGCGTATGCTCCGTAATG-3′	Annealing: 61°C	
Cx43	Forward: 5′-TACCACGCCACCACCGGCCCA-3′	30 cycles	293 bp
	Reverse: 5′-GGCATTTTGGCTGTCGTCAGGGAA-3′	Annealing: 60°C	
Cx45	Forward: 5′-CCCCTGATTTGCTACTAA-3′	35 cycles	250 bp
	Reverse: 5′-AATGCTAAGATTGCCTACA-3′	Annealing: 52°C	
Cx40	Forward: 5′-ATGGGTGACTGGAGCTTCC-3′	36 cycles	255 bp
	Reverse: 5′-CACAAAGATGATCTGCAGTACCC-3′	Annealing: 52°C	
18S	Forward: 5′-CCATCCAATCGGTAGTAGCG-3′	22 cycles	150 bp
	Reverse: 5′-GTAACCCGTTGAACCCCATT-3′	Annealing: 59°C	

### Western immunoblot

Left ventricles were homogenized at 4°C in radioimmunoprecipitation assay lysis buffer supplemented with protease inhibitor cocktail (Roche, Switzerland). The tissue homogenates were centrifuged at 14,000 g for 10 min at 4°C. Protein concentration in the soluble fraction was determined by Bradford method. Protein samples (100 µg) were separated by 12% SDS-polyacrylamide gel and then transferred onto polyvinylidene difluoride membranes. Membranes were then washed, blocked, and probed overnight at 4°C with rabbit anti-Cx43 (1∶1000, Chemicon, USA), Cx45 (1∶1000, Chemicon, USA), Cx40 (1∶1000, Chemicon, USA), JNK (1∶2000, Cell Signaling Technology, USA), phospho-JNK (1∶1000, Cell Signaling Technology, USA), β-actin (1∶2000, Santa Cruz Biotechnology, USA) and mouse monoclonal anti-CSE antibody (1∶1000, Abnova, Taiwan) respectively, followed by secondary antibody for 2 h with a 1∶2000 dilution of HRP-conjugated, goat anti-rabbit IgG or goat anti-mouse IgG (Santa Cruz Biotechnology, USA). Membranes were washed and then incubated in SuperSignal™ West Pico chemiluminescent substrate (Pierce, USA) before exposure to X-ray films (Pierce, USA). The intensity of bands was quantified using LabWorks™ Image Analysis software (UVP).

### Myeloperoxidase estimation (MPO)

Heart samples were thawed, homogenized in 20 mM phosphate buffer (pH 7.4), centrifuged (13,000× g, 10 min, 4°C) and the resulting pellets were resuspended in 50 mM phosphate buffer (pH 6.0) containing 0.5% w/v hexadecyltrimethylammonium bromide (Sigma). The suspension was subjected to four cycles of freezing and thawing and further disrupted by sonication (40 seconds). The samples were then centrifuged (13,000× g, 5 min, 4°C) and the supernatants were used for the MPO assay. The reaction mixture consisted of the supernatant (50 µl), 1.6 mM tetramethylbenzidine (Sigma), 80 mM sodium phosphate buffer (pH 5.4), and 0.3 mM hydrogen peroxide (reagent volume: 50 µl). This mixture was incubated at 37°C for 110 sec. The reaction was terminated with 50 µl of 0.18 M H2SO4 and the absorbance was measured at 405 nm. This absorbance was then corrected for the DNA content of the tissue sample and the results were expressed as enzyme activity.

### Assay of MDA content, activity of SOD and GSH-Px in heart

Cardiac tissue samples (25 mg) were homogenized in 0.5 ml of PBS at 4°C with a homogenizer. Homogenates were then centrifuged at 1500 rpm for 30 min and the supernatants were collected. MDA content as well as SOD and GSH-px activity in supernatants were evaluated with MDA, SOD and GSH-Px assay kits according to the manufacturer's protocol.

### Assay of creatine kinase (CK) and lactate dehydrogenase (LDH) enzyme activities

Plasma CK and LDH enzyme activities were measured using an autoanalyzer (Model 7020, Hitachi Medico, Japan).

### Transmission electron microscopy

In order to analyze gap junction remodeling, left ventricular tissues were immersion-fixed in phosphate-buffered 2.5% glutaraldeyde (pH 7.4), post-fixed with 1% osmium tetroxide, dehydrated through a graded ethanol series and embedded in Epon medium. Ultrathin sections were observed by electron microscopy (JEM1230; JEOL Transmission Electron Microscope, Tokyo, Japan). Five hearts from each group were examined. Ten pictures were taken from each heart and analyzed morphometrically using methods described previously [34], [35]. At a magnification of 30,000, every intercalated disk profile identified within each test area was rephotographed and the total number and length of gap junctions and the total length of intercalated disks were measured. Gap junction number and length was calculated per unit intercalated disk length as previously described [34], [35].

### Echocardiography

Mice were lightly anesthetized with isoflurane gas. AUBM system (Vevo 770, Visualsonics, Toronto, Canada) equipped with a 7.5-MHz imaging transducer was used for all the examinations. The transducer was placed in gentle contact with the mid-precordial area through a transmission medium. Left ventricular ejection fraction (LVEF), left ventricular fractional shortening (LVFS), left ventricular internal dimensions at the end of diastole (LVEDD) and at the end of systole (LVESD) were measured digitally on the M-mode tracing.

### Statistics

The data were expressed as mean ± SEM. The significance of differences among groups was evaluated by analysis of variance (ANOVA) with post-hoc Tukey's test when comparing three or more groups. The significance of differences between two groups was evaluated by T test. The overall survival analysis for lethal injury was described by Kaplan-Meier plots. The difference of mortality among groups was tested by log rank test. A P<0.05 was regarded as statistically significant.

## References

[1] Minotti G, Menna P, Salvatorelli E, Cairo G, Gianni L (2004). Anthracyclines: molecular advances and pharmacologic developments in antitumor activity and cardiotoxicity.. Pharmacol Rev.

[2] Hrdina R, Gersl V, Klimtová I, Simůnek T, Machácková J (2000). Anthracycline-induced cardiotoxicity.. Acta Medica (Hradec Kralove).

[3] Jones RL, Swanton C, Ewer MS (2006). Anthracycline cardiotoxicity.. Expert Opin Drug Saf.

[4] Wouters KA, Kremer LC, Miller TL, Herman EH, Lipshultz SE (2005). Protecting against anthracycline-induced myocardial damage: a review of the most promising strategies.. Br J Haematol.

[5] Singal PK, Iliskovic N, Li T, Kumar D (1997). Adriamycin cardiomyopathy: pathophysiology and prevention.. FASEB J.

[6] Łowicka E, Bełtowski J (2007). Hydrogen sulfide (H_2_S) – the third gas of interest for pharmacologists.. Pharmacol Rep.

[7] Su YW, Liang C, Jin HF, Tang XY, Han W (2009). Hydrogen sulfide regulates cardiac function and structure in adriamycin-induced cardiomyopathy.. Circ J.

[8] Li L, Rossoni G, Sparatore A, Lee LC, Del Soldato P (2007). Anti-inflammatory and gastrointestinal effects of a novel H_2_S-releasing diclofenac agent.. Free Radical Biol Med.

[9] Wallace JL, Caliendo G, Santagada V, Cirino G, Fiorucci S (2007). Gastrointestinal safety and anti-inflammatory effects of a hydrogen sulfide-releasing diclofenac derivative in the rat.. Gastroenterology.

[10] Rossoni G, Sparatore A, Tazzari V, Manfredi B, Del Soldato P (2008). The hydrogen sulphide-releasing derivative of diclofenac protects against ischaemia–reperfusion injury in the isolated rabbit heart.. Br J Pharmacol.

[11] Kumar NM, Gilula NB (1996). The gap junction communication channel.. Cell.

[12] Desplantez T, Dupont E, Severs NJ, Weingart R (2007). Gap junction channels and cardiac impulse propagation.. J Membr Biol.

[13] Geimonen E, Jiang W, Ali M, Fishman GI, Garfield RE (1996). Activation of protein kinase C in human uterine smooth muscle induces connexin-43 gene transcription through an AP-1 site in the promoter sequence.. J Biol Chem.

[14] Baldridge D, Lernando F, Shin CS, Stains J, Civitelli R (2001). Sequence and structure of the mouse connexin45 gene.. Bioscience Rep.

[15] Petrich BG, Gong X, Lerner DL, Wang X, Brown JH (2002). c-Jun N-terminal kinase activation mediates downregulation of connexin43 in cardiomyocytes.. Circ Res.

[16] Teunissen BE, Bierhuizen MF (2004). Transcriptional control of myocardial connexins.. Cardiovasc Res.

[17] Beardslee MA, Lerner DL, Tadros PN, Laing JG, Beyer EC (2000). Dephosphorylation and intracellular redistribution of ventricular connexin 43 during electrical uncoupling induced by ischemia.. Circ Res.

[18] Schulz R, Gres P, Skyschally A, Duschin A, Belosjorow S (2003). Ischemic preconditioning preserves connexin 43 phosphorylation during sustained ischemia in pig hearts in vivo.. FASEB J.

[19] De Angelis A, Piegari E, Cappetta D, Marino L, Filippelli A (2010). Anthracycline cardiomyopathy is mediated by depletion of the cardiac stem cell pool and is rescued by restoration of progenitor cell function.. Circulation.

[20] Huang C, Zhang X, Ramil JM, Rikka S, Kim L (2010). Juvenile exposure to anthracyclines impairs cardiac progenitor cell function and vascularization resulting in greater susceptibility to stress-ionduced myocardial injury in adult mice.. Circulation.

[21] Kimura Y, Kimura H (2004). Hydrogen sulfide protects neurons from oxidative stress.. FASEB J.

[22] Whiteman M, Armstrong JS, Chu SH, Jia-Ling S, Wong BS (2004). The novel neuromodulator hydrogen sulfide: an endogenous peroxynitrite ‘scavenger’?. J Neurochem.

[23] Whiteman M, Li L, Kostetski I, Chu SH, Siau JL (2006). Evidence for the formation of a novel nitrosothiol from the gaseous mediators nitric oxide and hydrogen sulphide.. Biochem Biophys Res Commun.

[24] Bhatia M, Sidhapuriwala JN, Sparatore A, Moore PK (2008). Treatment with H_2_S-releasing diclofenac protects mice against acute pancreatitis associated lung injury.. Shock.

[25] Sidhapuriwala J, Li L, Sparatore A, Bhatia M, Moore PK (2007). Effect of S-diclofenac, a novel hydrogen sulfide releasing derivative, on carrageenan-induced hindpaw oedema formation in the rat.. Eur J Pharmacol.

[26] Zhang H, Zhi l, Moochhala SM, Moore PK, Bhatia M (2007). Hydrogen sulfide acts as an inflammatory mediator in cecal ligation and puncture induced sepsis in mice by up-regulating the production of cytokines and chemokines via NF-κB.. Am J Physiol Lung Cell Mol Physiol.

[27] Bhatia M, Wong FL, Fu D, Law HY, Moochhala SM (2005). Role of hydrogen sulfide in acute pancreatitis and associated lung injury.. FASEB J.

[28] Ling L, Bhatia M, Yizhun Z, Yichun Z, Raina DR (2005). Hydrogen sulfide is a novel mediator of endotoxic shock.. FASEB J.

[29] Zanardo RC, Brancaleone V, Distrutti E, Fiorucci S, Cirino G (2006). Hydrogen sulfide is an endogenous modulator of leukocyte-mediated inflammation.. FASEB J.

[30] Fiorucci S, Antonelli E, Distrutti E, Rizzo G, Mencarelli A (2005). Inhibition of hydrogen sulfide generation contributes to gastric injury caused by anti-inflammatory nonsteroidal drugs.. Gastroenterology.

[31] Zhao W, Zhang J, Lu Y, Wang R (2001). The vasorelaxant effect of H_2_S as a novel endogenous gaseous K_ATP_ channel opener.. EMBO J.

[32] Bass SE, Sienkiewicz P, Macdonald CJ, Cheng RY, Sparatore A (2009). Novel dithiolethione-modified nonsteroidal anti-inflammatory drugs in human hepatoma HepG2 and colon LS180 cells.. Clin Cancer Res.

[33] Labarca C, Paigen K (1980). A simple, rapid, and sensitive DNA assay procedure.. Anal Biochem.

[34] Saffitz JE, Green KG, Kraft WJ, Schechtman KB, Yamada KA (2000). Effects of diminished expression of connexin43 on gap junction number and size in ventricular myocardium.. Am J Physiol Heart Circ Physiol.

[35] Luke RA, Saffitz JE (1991). Remodeling of ventricular conduction pathways in healed canine infarct border zones.. J Clin Invest.

